# The *Fusarium* Mycotoxin Deoxynivalenol Can Inhibit Plant Apoptosis-Like Programmed Cell Death

**DOI:** 10.1371/journal.pone.0069542

**Published:** 2013-07-26

**Authors:** Mark Diamond, Theresa J. Reape, Olga Rocha, Siamsa M. Doyle, Joanna Kacprzyk, Fiona M. Doohan, Paul F. McCabe

**Affiliations:** School of Biology and Environmental Science, University College Dublin, Dublin, Ireland; Soonchunhyang University, Republic of Korea

## Abstract

The *Fusarium* genus of fungi is responsible for commercially devastating crop diseases and the contamination of cereals with harmful mycotoxins. *Fusarium* mycotoxins aid infection, establishment, and spread of the fungus within the host plant. We investigated the effects of the *Fusarium* mycotoxin deoxynivalenol (DON) on the viability of Arabidopsis cells. Although it is known to trigger apoptosis in animal cells, DON treatment at low concentrations surprisingly did not kill these cells. On the contrary, we found that DON inhibited apoptosis-like programmed cell death (PCD) in Arabidopsis cells subjected to abiotic stress treatment in a manner independent of mitochondrial cytochrome c release. This suggested that *Fusarium* may utilise mycotoxins to suppress plant apoptosis-like PCD. To test this, we infected Arabidopsis cells with a wild type and a DON-minus mutant strain of *F. graminearum* and found that only the DON producing strain could inhibit death induced by heat treatment. These results indicate that mycotoxins may be capable of disarming plant apoptosis-like PCD and thereby suggest a novel way that some fungi can influence plant cell fate.

## Introduction

*Fusarium* fungi cause some of the most commercially devastating diseases of rice, corn, barley, wheat and other food crops, and as a result world agriculture suffers massive produce loss each year. For example, *Fusarium* head blight (FHB) disease of wheat and barley caused direct and secondary economic losses of around $2.7 billion in the central United States between 1998 and 2000 [1], [2]. Much of the economic losses attributed to *Fusarium* sp. are not simply due to reduced crop yield but also because of the levels of mycotoxins produced by these fungi. A 2003 investigation on the occurrence of *Fusarium* mycotoxins led by the European Union (EU) Scientific Cooperation project showed that 61% of 6,358 wheat samples studied were contaminated with the *Fusarium* mycotoxin deoxynivalenol (DON) at levels often exceeding the maximal permissible limit of 1,750 µg/kg set by EU regulations [3].

DON has been shown to be important for the spread and establishment of *F. graminearum* within the host plant. Knockout mutants of *F. graminearum* in which the ability to produce DON is retarded, are able to infect, but not spread within the host plant [4], [5]. Many studies in animal systems have established a relationship between DON and programmed cell death (PCD), for example DON induced an apoptotic death when added to human intestinal and erythroleukemia cell lines [6], [7]. DON is a low molecular weight sesquiterpenoid epoxide trichothecene and a potent inhibitor of eukaryotic protein synthesis. DON blocks the production of proteins by binding to the 60S ribosomal subunit and inhibiting peptidyltransferase [8]. However, the PCD-inducing effects of DON might not be purely down to an arrest of protein synthesis [8]. For example, DON treatment of Jurkat human T-lymphoid cell lines resulted in the activation of a ribotoxic stress response and signalling cascade, which can also lead to apoptotic PCD [9].

In plants, PCD is activated in response to fungal, bacterial and viral pathogens. The host’s response to avirulent pathogens often terminates in the rapid death of infected or challenged cells, which can result in arrest of pathogen growth [10]. It is also thought that PCD has a role in promoting the growth of some pathogens, especially those that secrete toxins in order to kill host cells rapidly [11]. AAL, the toxin produced by the tomato pathogen *Alternaria alternata* f. sp. *Lycopersici,* induces PCD, and pathogens lacking the ability to produce this toxin have severely reduced growth on susceptible plants [12].

In this study the effects of DON treatment and *F. graminearum* infection on plant apoptosis-like PCD were assessed using an Arabidopsis cell culture system. Many of the fundamental breakthroughs in animal apoptosis have been made in simpler model systems, which include cell-free systems, cell cultures, and nematode research. For plant PCD research purposes, cell cultures have many advantages over whole plants [13]. Firstly, cells undergoing PCD are readily accessible in cell culture, whereas in whole plants PCD can occur deeply embedded within otherwise healthy tissue (e.g. in tapetal cells). Secondly, it is easier to quantify the numbers of viable cells in a cell culture through the use of vital stains such as fluorescein diacetate (FDA). Thirdly, cell cultures are more amenable for testing the effects of certain compounds/drugs as it is possible to add the compound, monitor its effects over time by extracting small samples, and be sure that all of the cells in the culture are subjected to similar concentrations. In our Arabidopsis cell culture model an apoptosis-like PCD can be distinguished from other forms of plant cell death by protoplast condensation that results in a morphologically distinct cell corpse [14], [15]. The higher the stress, the higher the proportion of cells which die via necrosis as opposed to apoptosis-like PCD [16]. This is significant when treating cells with compounds as it is crucial to be able to not just observe viability but distinguish between different forms of death, e.g. apoptosis-like PCD and necrosis, as the level of stress that a cell is exposed to can be a critical determinant on the ultimate fate of the cell [16], [17].

Here we report that DON, a mycotoxin produced by the hemibiotrophic pathogen *F. graminearum*, inhibits apoptosis-like PCD induced by heat stress and ethanol treatment. We also show that inhibition of apoptosis-like PCD occurs despite the release of mitochondrial cytochrome c. Furthermore, by infecting cell cultures with either a DON-producing strain or a DON-minus strain of *F. graminearum*, we have demonstrated that the fungus can also inhibit apoptosis-like PCD induced by heat treatment but only when it has the capability to produce the mycotoxin. To our knowledge, this is the first documented incidence of fungal-produced proteins or mycotoxins inhibiting plant apoptosis-like PCD. That this inhibition occurs in cell cultures of a plant susceptible to infection by the fungus [18] suggests that inhibition of apoptosis-like PCD may play an important part in the plant colonisation process of some fungi.

## Materials and Methods

### Cell Growth Conditions

Cells were grown in liquid Murashige and Skoog (MS) medium [19], [13]. Cells were sub-cultured by pipetting 12 ml of culture into 100 ml of fresh medium every 7 days, and were grown on a rotary shaker at 110 rpm (5 cm rotation) under a constant light (6 µmol m^−2 ^s^−1^) and temperature regime at 22°C.

### Chemical Treatment of Whole Cells

DON (Sigma) was dissolved in water at a concentration of 2000 ppm and stored at 4°C. Cells were treated with 10 ppM DON 24 hours prior to ethanol or heat treatment. Cycloheximide (CHX) (Sigma) was dissolved in water at a concentration of 5 mg/ml and stored at 4°C. Cells were treated with 100 µg/ml CHX 24 hours prior to heat treatment. Cells were treated with 10% (v/v) ethanol (Merck) and incubated under normal growth conditions.

### Heat Treatment of Cells

For heat treatment, 10 ml of cells were transferred to sterile 100 ml conical flasks and placed in a water bath with an orbital shaker at 90 rpm that was preheated to 53°C. Samples were treated for 13 minutes and were then removed to normal growth conditions. After 4–5 hours cells were scored for viability and morphology.

### Scoring Cells

**Figure 1:**
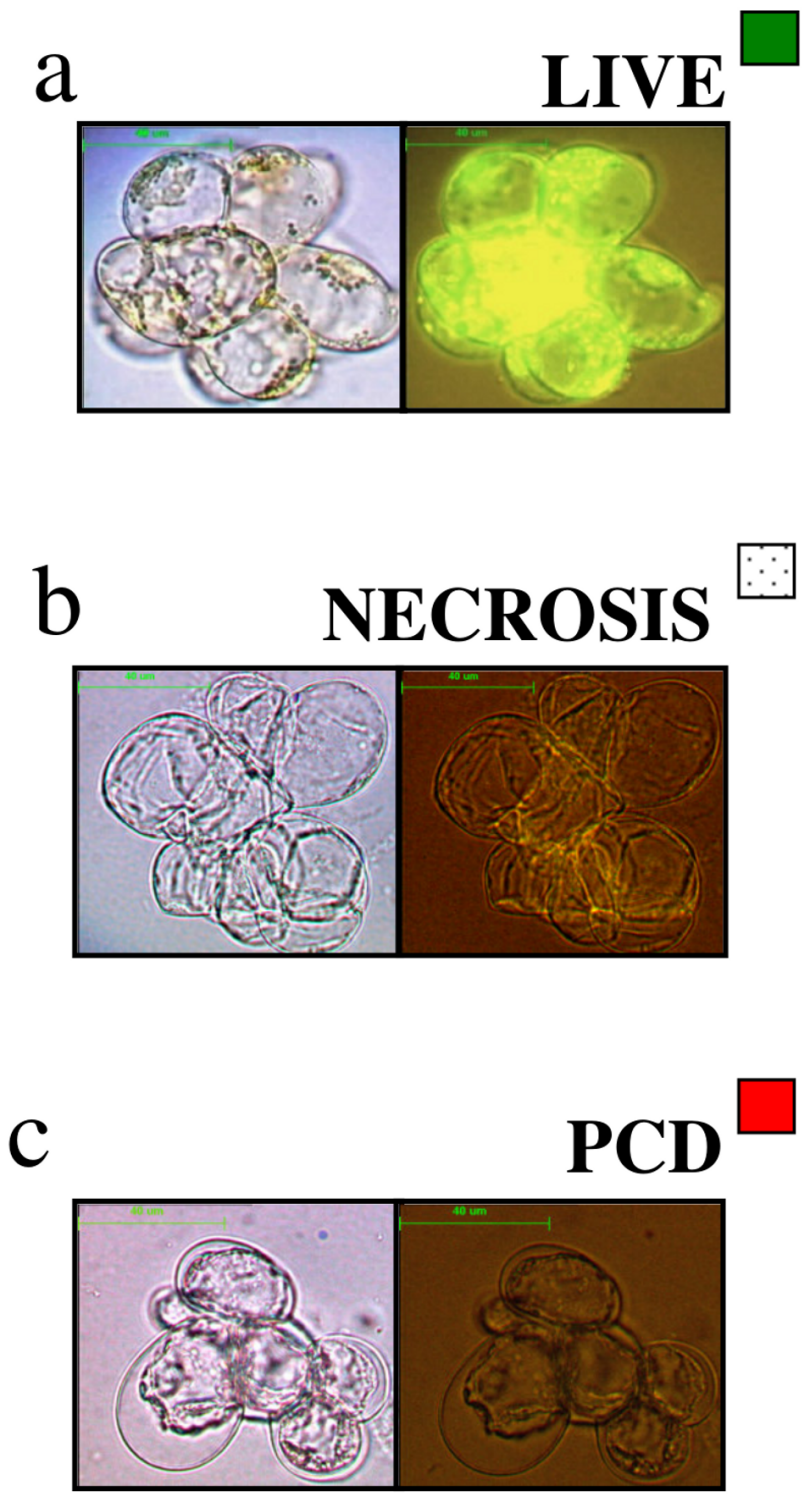
Cell types present following a 10 minute 53°C heat treatment. (a) Cells that are alive have the ability to cleave FDA, and fluoresce under light at a wavelength of 490 nm. (b) Necrotic cells cannot cleave FDA so do not fluoresce and show no evidence of protoplast condensation. (c) In cells that undergo apoptosis-like PCD the protoplast retracts from the cell wall. These cells cannot cleave FDA, so do not fluoresce. As this is a moderate stress, the majority of the cells die via apoptosis-like PCD.

Cells were examined under a phase contrast microscope (Leica DMLB) with an attached fluorescence lamp and camera. The vital stain fluorescein diacetate (FDA) was used to assay for live cells [13]. When FDA is excited by light at a wavelength of 490 nm, a bright green fluorescence is observed in viable cells whose plasma membrane (PM) is intact (Figure 1a). Cells that die by necrosis do not display the protoplast retraction associated with apoptosis-like PCD and do not fluoresce (Figure 1b). Cells that have undergone apoptosis-like PCD (Figure 1c) show a characteristic retraction of the protoplast away from the cell wall and do not fluoresce.

### Inoculation of Cells with Conidiospores

Conidia of *F. graminearum* mutant (GZT40) and wild-type (GZ3639) strains were produced as described previously [20]. Arabidopsis cell samples (10 ml) were transferred to sterile 100 ml conical flasks and were treated with 2 ml of either conidial inoculum (5×10^4^/ml) or water. Samples were then maintained in an incubator with an orbital shaker at 110 rpm, in the dark at a constant 22°C.

### Isolation of Mitochondria from Cells

All steps were performed at 4°C unless otherwise stated. Heat shocked or control (untreated) Arabidopsis suspension cells (200 ml) were filtered through one layer of Miracloth and then homogenised using a mortar and pestle in 0.3 M mannitol, 50 mM sodium pyrophosphate, 0.2% (w/v) BSA, 0.5% (w/v) polyvinylpyrrolidone-40, 2 mM EGTA, 20 mM cysteine, pH 8.0/phosphoric acid and glass beads for approximately 3×4 minutes or until ∼80% of cells were disrupted, filtering through 2 layers of Miracloth each time and adding fresh buffer. Extracts were centrifuged at 2,000×g for 10 minutes to remove debris and the supernatant centrifuged at 13,000×g for 15 minutes. The supernatant (cytosolic fraction) was stored for further analysis. The crude mitochondrial pellet was re-suspended in 0.3 M mannitol, 10 mM TES-KOH, pH 7.5 (wash buffer) and homogenised briefly using a Potter-Elvehjem pestle and glass tube to remove any clumps, centrifuged until the rotor reached ∼7,000×g and then re-centrifuged at 13,000×g for 15 minutes. The resuspended pellet was re-homogenised to remove clumps, loaded onto a 40, 23 and 18% percoll step gradient (in 0.3 M mannitol, 10 mM TES, pH 7.5 and 0.1% BSA) and centrifuged at 40,000×g for 45 minutes with the brake off. The purified mitochondria were collected at the 23 to 40% interface and washed several times in wash buffer. Mitochondria were finally re-suspended in wash buffer and immediately following protein determination (Bradford method, Biorad), 2× SDS loading buffer (100 mM Tris-HCL, pH 6.8, 4% SDS, 40% glycerol and 200 mM fresh DTT) was added and samples stored at –70°C prior to Western blot analysis.

### Western Blot Analysis

Protein (40 µg) was separated on a 15% SDS-PAGE gel [21], transferred to nitrocellulose (Optitran BA-S 83, 0.2 µm, Schleicher and Schuell), blocked with 10% dried milk/0.1% Tween 20/PBS, incubated with antibodies to cytochrome c 7H8.2C12 (BD Pharmingen, Oxford, UK) or VDAC/porin (Sigma) at 1∶1000, washed and then incubated with HRP-conjugated anti-mouse (Pierce) or rabbit IgG (Sigma), respectively. Labelled proteins were detected using chemiluminescence (Pierce Biotechnology, Rockford, IL, USA) following manufacturer’s instructions.

### Statistical Analysis

Data presented were transformed to follow normal distribution using Johnson transformation [22] and treatments were compared using ANOVA incorporating Tukey’s pairwise comparison test (family error rate  = 0.05). Data presented that could not be transformed to follow a normal distribution were compared using the Mann-Whitney 2-sample rank test. All analyses were conducted using Minitab® release 14 (Minitab Ltd., UK).

## Results

### DON Inhibits Apoptosis-like PCD Induced by Heat Shock and Ethanol Treatment

**Figure 2:**
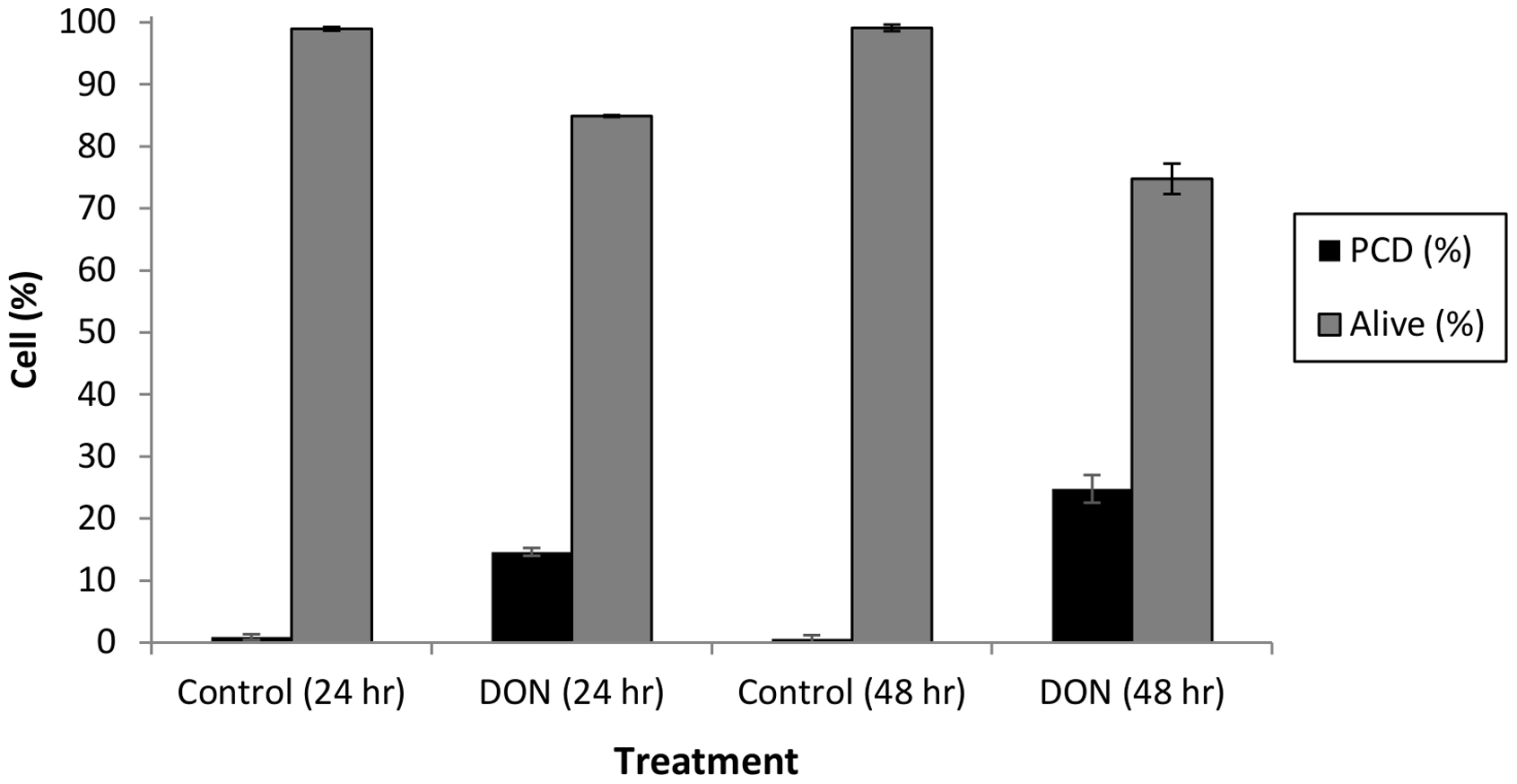
The effect of a high concentration of DON on viability of Arabidopsis suspension cells. Arabidopsis suspension cells were treated with either water control or 120 ppm DON for 24 and 48 hours. Apoptosis-like PCD increased at 24 and 48 hours. Results are the mean percentage (+/− standard deviations) of cells in a given state, taken from three independent experiments each carried out in triplicate.

DON has been shown to trigger apoptosis when added to animal cells [6], [7]. We investigated the effects of similar concentrations of DON on plant cells. Cell cultures [19] of an Arabidopsis genotype (Landsberg *erecta*), that are susceptible to DON-producing *Fusaria* [18], were incubated in a range of DON concentrations (1 to 40 ppm). Over the next 48 hours cell viability was scored with the vital stain fluorescein diacetate (FDA) and apoptosis-like PCD was assessed by the shrunken corpse morphology (see materials and methods) that has been shown to be characteristic of apoptosis-like PCD in plant cells [23]–[25]. No significant changes in death levels were found under any treatment in this time range (results not shown). So, unlike the situation in animal cells, DON does not induce apoptosis-like PCD in Arabidopsis cells at lower concentrations during this time period. However, at a concentration of 120 ppm, DON induced death, with 24.8% of the cells having undergone apoptosis-like PCD after 48 hours (Figure 2).

**Figure 3:**
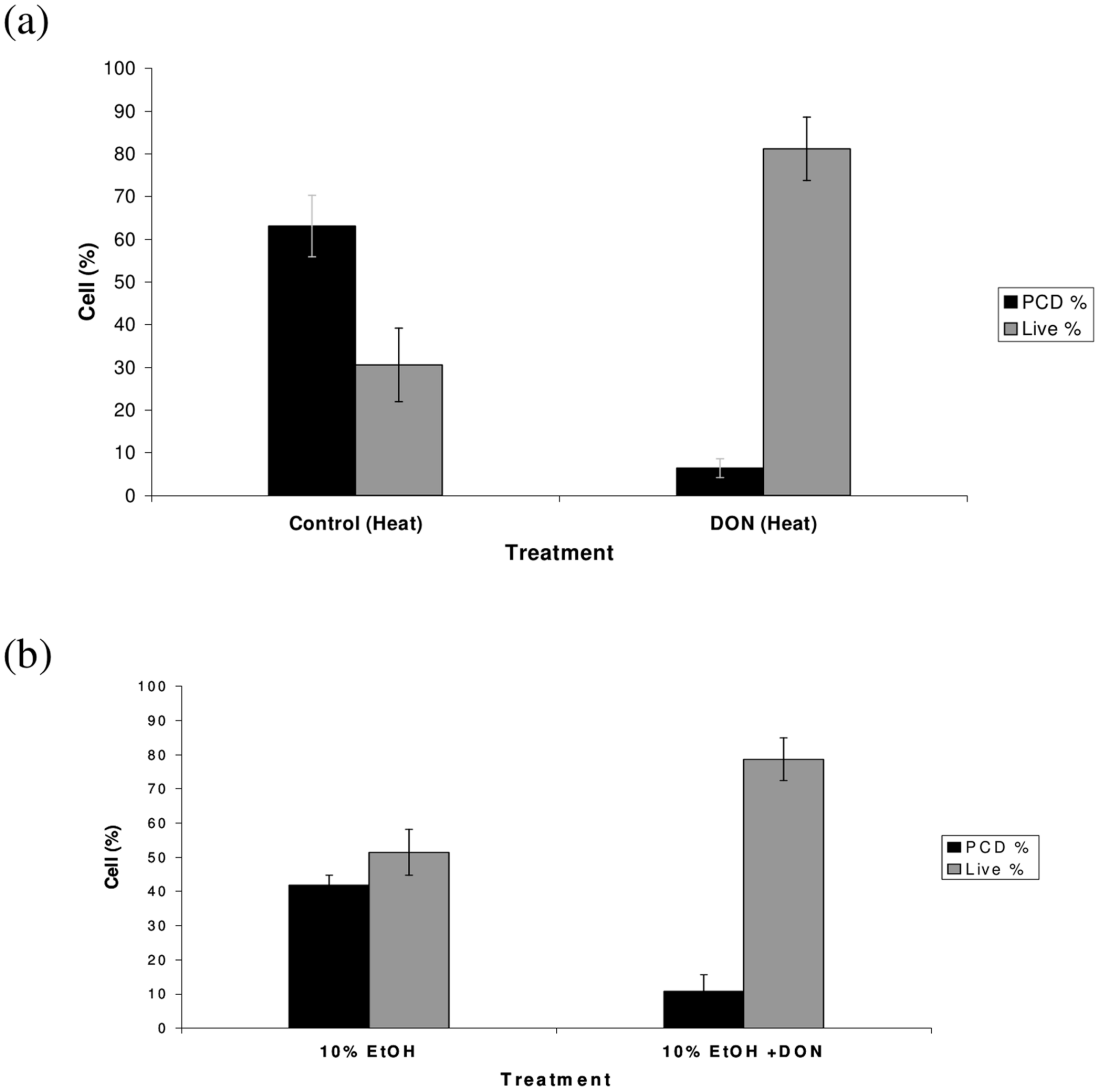
The effect of DON on heat and ethanol induced apoptosis-like PCD. (a) Heat treatment caused a high level of apoptosis-like PCD in control samples. In samples that were treated with DON, apoptosis-like PCD was greatly reduced, while the number of live cells increased. Results are the mean percentage (+/− standard deviations) of cells in a given state, taken from two independent experiments each carried out in triplicate. (b) Ethanol (EtOH) treatment induced apoptosis-like PCD in control samples. In samples that were treated with DON, apoptosis-like PCD was reduced, while the number of live cells increased. Results are the mean percentage (+/− standard deviations) of cells in a given state, taken from two independent experiments each carried out in triplicate.

We next investigated if DON, at lower concentrations, could, rather than inducing PCD, interfere with the cell death process in plants. To test this we investigated the effect of DON on heat stress-induced apoptosis-like PCD in Arabidopsis cell cultures. Short heat treatments are effective initiators of the cell death programme in cultured plant cells [23], [25], including Arabidopsis [13], [24]. Cells were incubated with DON at a concentration of 10 ppm (34 µM), 24 hours prior to heat treatment. The effects of DON on heat-induced apoptosis-like PCD can be seen in Figure 3a. In control samples, 5 hours after heat treatment, 30.6% of cells remained alive, while 63.1% died and displayed the shrunken morphology indicative of apoptosis-like PCD. In contrast, cells that were pre-incubated with DON, showed a marked difference to control cells; 81.2% of these cells were alive, an increase of more than 2.5-fold on the control (*P*  = 0.001). Only 6.4% of DON-treated cells died from apoptosis-like PCD, almost a 10-fold decrease compared to control cells (*P*  = 0.001). This demonstrates that DON strongly inhibits cell death activation for at least the first 5 hours after a PCD-inducing stress.

In order to test whether DON blocks apoptosis-like PCD triggered by an alternative inducer, we investigated the effects of DON on ethanol-induced PCD (previously shown to induce PCD in carrot cells [23]). Cells were incubated with DON at a concentration of 10 ppm, 20 hours prior to ethanol (10% v/v) treatment (Figure 3b). In ethanol-treated samples, 4 hours after treatment, 51.5% of cells remained alive, while 41.9% died and displayed the shrunken morphology indicative of apoptosis-like PCD. In contrast, 78.7% of cells that were pre-incubated with DON remained alive. Only 10.8% of DON-treated cells died from apoptosis-like PCD, almost a 4-fold decrease compared to control cells (*P*  = 0.005). These results demonstrate that DON can partially inhibit the death induced by ethanol treatment.

**Figure 4:**
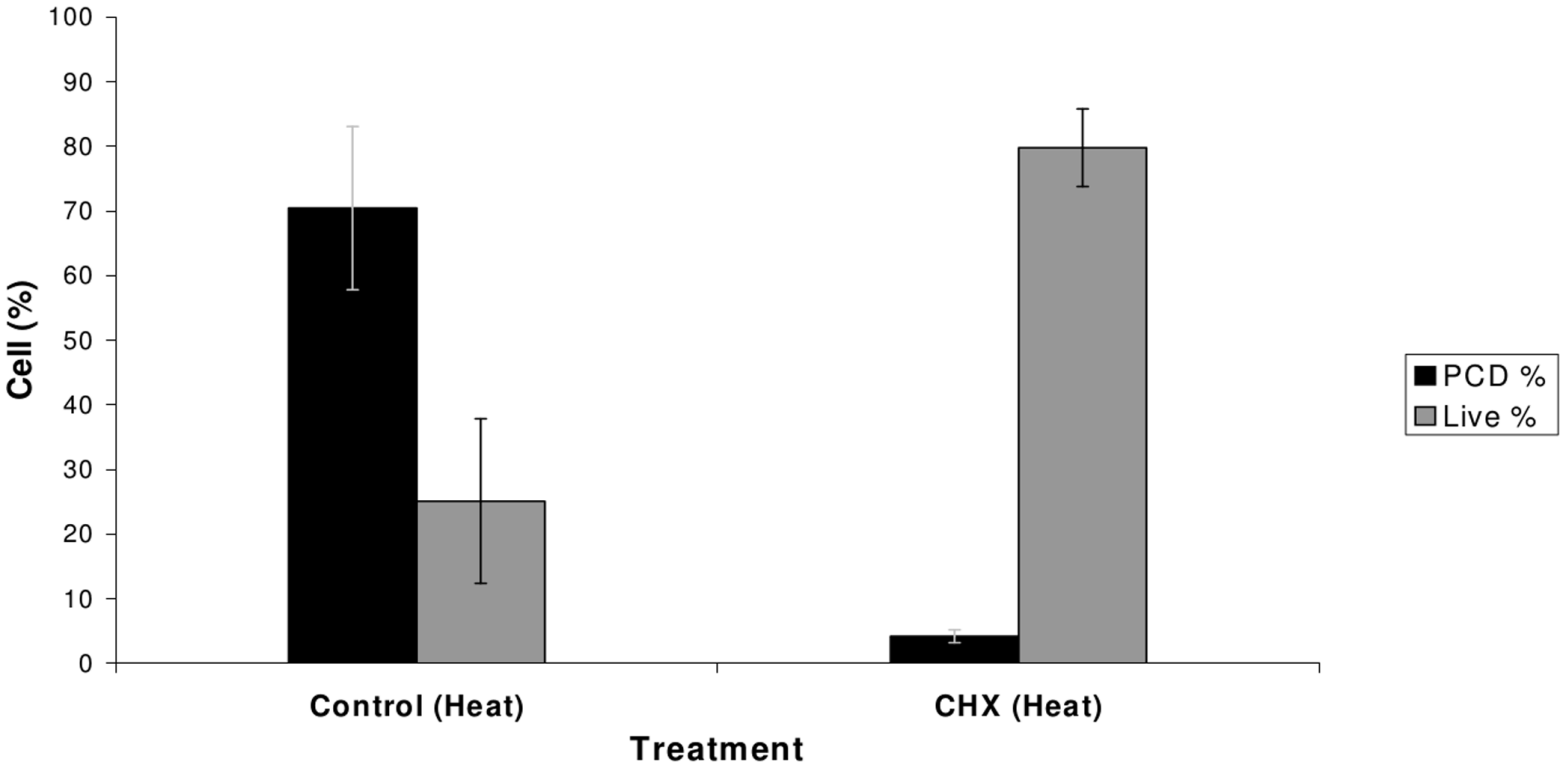
The effect of CHX on heat-induced apoptosis-like PCD. Heat treatment caused a high level of apoptosis-like PCD in control samples. In samples treated with CHX, apoptosis-like PCD was greatly reduced, while the number of live cells increased. Results are the mean percentage (+/− standard deviations) of cells in a given state, taken from two independent experiments each carried out in triplicate.

In order to test whether an alternative protein synthesis inhibitor, cycloheximide (CHX), had similar protective effects to DON, we repeated the heat stress experiment in the presence of CHX. At 6 hours, 25.1% of control (no CHX) heat-treated cells remained alive (Figure 4), while 70.5% died through apoptosis-like PCD. In cells incubated with 100 µg/ml CHX (355 µM) prior to heat treatment, apoptosis-like PCD was reduced to 4.2% (*P*  = 0.001) and the percentage of live cells increased to 79.8% (*P  = *0.001). This demonstrates that like DON, CHX can strongly inhibit apoptosis-like PCD induced by heat treatment.

### DON does not Inhibit Mitochondrial Cytochrome c Release

**Figure 5:**
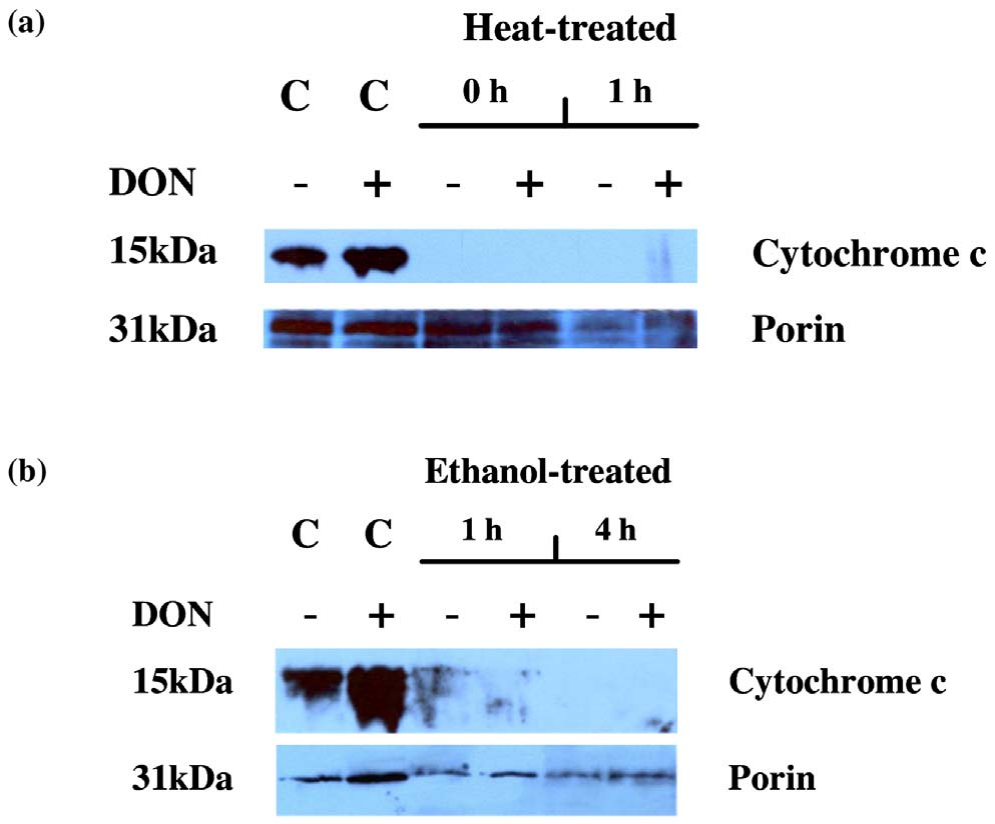
The effect of DON on heat and ethanol induced mitochondrial cytochrome c release. (a) Cytochrome c (Cyt c) remained associated with the mitochondrial fraction of non-heat-treated control cell samples (+/−DON). However, Cyt c release from the mitochondrial fraction was induced immediately following a 10 minute heat treatment (0 h +/− DON). VDAC/porin remained associated with the mitochondrial fraction in all samples. C = control samples. (b) Cyt c remained associated with the mitochondrial fraction of non-EtOH-treated control cell samples (+/−DON). Mitochondrial Cyt c release occurred in all samples treated with EtOH (+/− DON) at 1 hour and 4 hours post EtOH treatment. VDAC/porin remained associated with the mitochondrial fraction in all samples. C = control samples.

It has been suggested that DON-related protein synthesis inhibition directly triggers apoptosis in animal cells [7]. The intrinsic pathway of apoptosis is driven by cytochrome c release from the mitochondrion and subsequent activation of cytoplasmic caspases that systematically dismantle the animal cell. Because cytochrome c only needs to be translocated from the mitochondrion to activate apoptosis, and inactive caspases are constitutively present in the cytoplasm, apoptosis can proceed independently of the need for *de novo* protein production. In plant cells, cytochrome c release has been shown to occur in response to a wide range of PCD-inducing stimuli [14], [15]. We investigated if DON inhibits the release of cytochrome c, as this would suggest a possible mode of action by which it could block PCD. Heat treatment has been shown previously to trigger cytochrome c release in Arabidopsis cells [26]. Therefore we investigated the effects of DON on heat-induced cytochrome c release (Figure 5a). Cells were incubated with DON (10 ppm) 24-hours prior to heat treatment (53°C). Mitochondria were isolated from cells either immediately or 1 hour following a 10 minute heat treatment. Cytochrome c was present in non-heat-treated control cells (+/−DON), but could not be detected in the mitochondrial fraction of heat-treated cells (+/−DON). Additionally, VDAC/porin, an integral protein of the outer mitochondrial membrane, remained associated with the mitochondrial fraction in control samples and in heat-treated (+/− DON) samples isolated at 0 hours. This data indicates that DON did not inhibit the migration of cytochrome c induced by heat treatment and that the outer mitochondrial membrane remained intact during the period of cytochrome c release. Cytochrome c could not be detected in cytosolic fractions. Similar results were obtained with ethanol treatment, with cytochrome c present in untreated and DON-treated cells, but absent in the mitochondrial fraction of ethanol-treated cells (+/− DON) at 1 hour, or 4 hours following ethanol treatment (Figure 5b). As with heat shock experiments, cytochrome c could not be detected in cytosolic fractions.

### *Fusarium graminearum* Infection Interferes with Apoptosis-like PCD Induction

**Figure 6:**
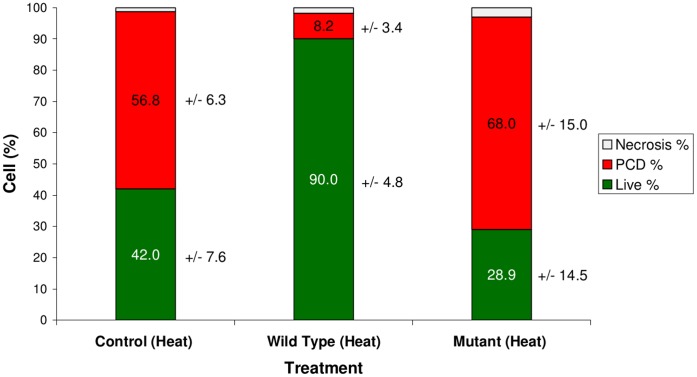
The effect of infection with wild-type and DON-minus strains of *F. graminearum* on heat-induced apoptosis-like PCD in Arabidopsis cells. Heat treatment caused a high level of apoptosis-like PCD in control samples and few live cells were observed. Samples that were inoculated with DON-minus mutant (GZT40) spores had decreased viability, and apoptosis-like PCD was increased in comparison to controls. Samples that were inoculated and infected with ‘wild type’ (GZ3639) spores had a much-reduced level of apoptosis-like PCD and cell death in general, while the number of live cells was increased. Results are the mean percentage (+/− standard deviations) of cells in a given state, taken from two independent experiments each carried out in triplicate.

The action of DON in this study raises the possibility that a general role for mycotoxins may be to disable plant PCD responses. This suppression of PCD together with the evidence that the absence of DON restricts fungal spread in host tissue [4], [5] strongly suggests that PCD can compromise *Fusarium* fungal infection. To investigate this possibility, we tested if the DON-producing *F. graminearum* strain GZ3639 could also inhibit apoptosis-like PCD caused by heat treatment. Furthermore, we included a trichothecene-minus mutant (strain GZT40) of the fungus [4], [5] in the experiment (Figure 6).

Twenty hours post-inoculation of cell cultures, the spores of both the GZ3639 and GZT40 strains had germinated and infected the majority of cell clusters. No differences in either the growth of the two fungal strains or the successful infection of the host Arabidopsis cells were noted at this time. This was therefore taken as an appropriate time point for heat treatment, and cells were subsequently scored for apoptosis-like PCD morphology and viability 4 hours later. Total cell death in mutant (GZT40)-inoculated heat-treated cultures was significantly higher than that in non-inoculated heat-treated cultures (71.1 and 58%, respectively) (*P*>0.05). In both treatments, the majority of dead cells had activated AL-PCD (68 and 56.8%, respectively). In contrast, total death was reduced to 10%, and apoptosis-like PCD was reduced to 8.2% in cultures treated with ‘wild type’ (GZ3639) conidiospores. These results demonstrate that DON production is essential for *F. graminearum* to successfully inhibit heat-induced apoptosis-like PCD in Arabidopsis cells.

## Discussion

We show that the mycotoxin DON (at low concentrations) and the protein synthesis inhibitor CHX can block apoptosis-like PCD. While CHX has been shown previously to inhibit heat-induced cell death [27], [28] it could not be presumed that DON would have such an effect. Firstly, DON is a known virulence factor that aids the spread of disease. Knockout mutants of *F. graminearum* in which the ability to produce DON is retarded, are able to infect, but not spread within the host plant [4], [5]. In contrast, the genus of bacteria which produce cycloheximide, *Streptomyces*, are largely known for their saprotrophic activity in soil or their endopyhtic anti-fungal activity in plant roots [29]. Secondly, DON and CHX differ in their eukaryotic ribosomal protein targets and also differ in the effects they have on intracellular signalling pathways in animal cells. The ribosomal target of DON in yeast is the L3 protein, whereas CHX is known to target the yeast L29 ribosomal protein [30], [31]. DON also strongly induces the activation of a stress activated protein kinase (SAPK/JNK1) in mammalian cells within 15 minutes of treatment, whereas CHX does not [32], [8], [33]. Further evidence that the effects of DON may not be purely due to its protein synthesis inhibition activity was provided by Desmond et al., (2008) [34]. The authors demonstrated that DON actually induces the production of a suite of defence gene transcripts and proteins in wheat seedlings, including chitinase (PR2) and beta-1,3-glucanase (PR3).

Because it has been shown that DON triggers apoptosis in animal cells [7] we decided to investigate if DON affects the release of cytochrome c from the mitochondrion, as this would suggest a possible mode of action by which it could block apoptosis-like PCD. The results however indicated that cytochrome c release induced by different death stimuli is not affected by the survival-promoting activity of DON. This is suggestive of a divergent role for cytochrome c in plant apoptosis-like PCD. While in animal cells it is known that cytochrome c release occurring at the onset of PCD can lead to the activation of cytoplasmic PCD proteins, no such proteins have been characterised in plants. Animal cytochrome c has been shown to activate molecules with a caspase-like activity in carrot cytoplasm which could then degrade rat liver nuclei in an apoptotic fashion [35]. However, C_2_ ceramide-induced death of tracheary element (TE) cells in *Zinnia elegans* was found to be independent of cytochrome c release [36]. The authors of this study showed that inhibiting mitochondrial permeability transition with cyclosporine A could block TE death, however this inhibition did not result in the blocking of cytochrome c release. This result suggests that cytochrome c relocation was insufficient to trigger PCD in those cells. Additionally Balk et al., (2003) [26] were unable to activate apoptosis-like PCD by adding purified cytochrome c to an Arabidopsis cell-free system. The results of our study further demonstrate that cytochrome c release may not directly lead to apoptosis-like PCD in plant cells. We have shown that Arabidopsis cells challenged with heat remain viable during a time period when cytochrome c is no longer detectable in the mitochondria. We were unable to find any cytochrome c associated with our cytosolic fractions using our detection system and we have previously found this to be the case in Arabidopsis cells [26]. This is not surprising given the findings of Vacca et al, (2006) [28], where cytochrome c was found to be degraded in a caspase-dependent manner during heat shock-induced cell death of tobacco cells. While cytochrome c release does not appear to directly activate apoptosis-like PCD, the results obtained in this study suggest that the *de novo* synthesis of proteins may be required for the execution of apoptosis-like PCD in higher plants, at least in response to heat stress.

While traditionally regarded as a necrotroph, there is increasing evidence and belief that *F. graminearum* is actually a hemibiotrophic pathogen, with a short biotrophic phase preceding the nectrophic phase of disease spread [37]. DON suppression of death suggests that the role of DON may be to disable PCD during the initial biotrophic infection stages in plant cells. To investigate this possibility we infected Arabidopsis cells with a DON-producing strain and a DON-minus strain of *F. graminearum*. The results indicate that *F. graminearum* infection can inhibit heat-induced apoptosis-like PCD in Arabidopsis cells, however this inhibition only occurred when the cells were infected with the DON-producing strain (GZ3639). Since strain GZ3639 was ultimately more successful at spreading through and killing the Arabidopsis cells (data not shown), these results reaffirm an important role for DON as a virulence factor and suggest that virulence is enhanced by the inhibition of plant apoptosis-like PCD. Cuzick et al. (2008) [38] found that a trichothecene-minus mutant of *F. graminearum* was as pathogenic to Arabidopsis floral organs as was the wild type. Thus, effects of DON on cellular virulence may not manifest as disease spread through Arabidopsis tissue.

Recent studies carried out with wheat and barley suggest contrasting roles for DON during *Fusarium* infection of host plants, which are dependent on treatment duration and DON concentration. In wheat, Desmond et al. (2008) [33] demonstrated that infusion of leaves with 100–200 ppm DON caused H_2_O_2_ production within 6 hours and cell death (identified by trypan blue staining and observation of DNA laddering) within 24 hours. However, previous work by Mudge et al., (2006) [39] had shown that such high concentrations of DON only occur during *F. graminearum* infection of wheat at late necrotrophic stages of infection (14–28 days). Therefore, Desmond and co-workers (2008) [33] acknowledged that the PCD response of the host to DON is associated with the later stages of crown rot disease, when the host has developed visible lesions. In barley leaf segments Bushnell et al. (2010) [40] demonstrated that high concentrations of DON (90 ppm) bleached some treated tissues and that this effect could be enhanced by the addition of 1–10 mM Ca^2+^. At lower concentration (10 ppm), DON, by itself, did not have any bleaching effect. However the authors also showed that some leaf segments remained greener than tissues in control segments through 72 hours, when treated with DON (at 30 ppm and 90 ppm). This suggests that DON could have dual effects in delaying the chlorosis associated with senescence and perhaps contributing to cell death during FHB when it accumulates to high concentrations. Taken together, these studies and ours suggest that DON can have dual roles during the colonisation of plant tissue by *F. graminearum.* At lower concentrations and during early biotrophic stages of infection, DON could inhibit host cell PCD facilitating the initial spread of the fungus. However, at a later necrotrophic stage of infection, higher DON concentrations could actually bring about host cell death and generate a source of nutrition for the pathogen.

To our knowledge, this is the first incidence of a fungal mycotoxin inhibiting plant apoptosis-like PCD. There are incidences of bacterial factors inhibiting PCD in plants and in animals. For example, the pathogenic bacterium *Pseudomonas syringae* produces several factors that are capable of inhibiting PCD in plants and yeast [41]. These factors are translocated into the plant cell via the bacterial type III secretion system and were found to effectively suppress the hypersensitive response in tobacco and Arabidopsis. Animal pathogens have also been shown to have anti-apoptotic abilities. The obligate intracellular bacterium *Chlamydia pneumoniae,* which causes airway infections in humans, is known to synthesise proteins that interfere with the host cell’s PCD mechanism [42]. The hemi-biotroph *Cladosporium fulvum* grows through the intercellular space in tomato without inducing the hypersensitive response or PCD [43]. It is now known that infection by the fungus induces the production of γ-aminobutyric acid (GABA), which has a proposed role in protecting the plant from oxidative stress [44].

In conclusion, we have demonstrated that the capacity of *Fusarium* to produce DON affects plant PCD. From our work and the work of others we now know that relatively low concentrations of DON inhibit PCD, while high concentrations induce cell death. The question now arises as to whether the role of DON in disease is much more refined than previously thought. DON has been shown to move into host plant tissue in advance of the colonising fungus, and the production of DON is a requirement for the spread of the fungus [5], [42]. But at the initial infection point, it is conceivable that apoptosis-like PCD can jeopardise biotrophism and that DON is recruited by the pathogen to interfere with PCD and the associated cascade of defence responses.
